# Supplementary material for: Measuring compound eye optics with microscope and microCT images
Source: Commun Biol. 2023 Mar 7;6:246. doi: 10.1038/s42003-023-04575-x (PMC9992655; doi:10.1038/s42003-023-04575-x)
Supplement: Supplementary file 2 — Supplementary Information [file 42003_2023_4575_MOESM2_ESM.pdf]

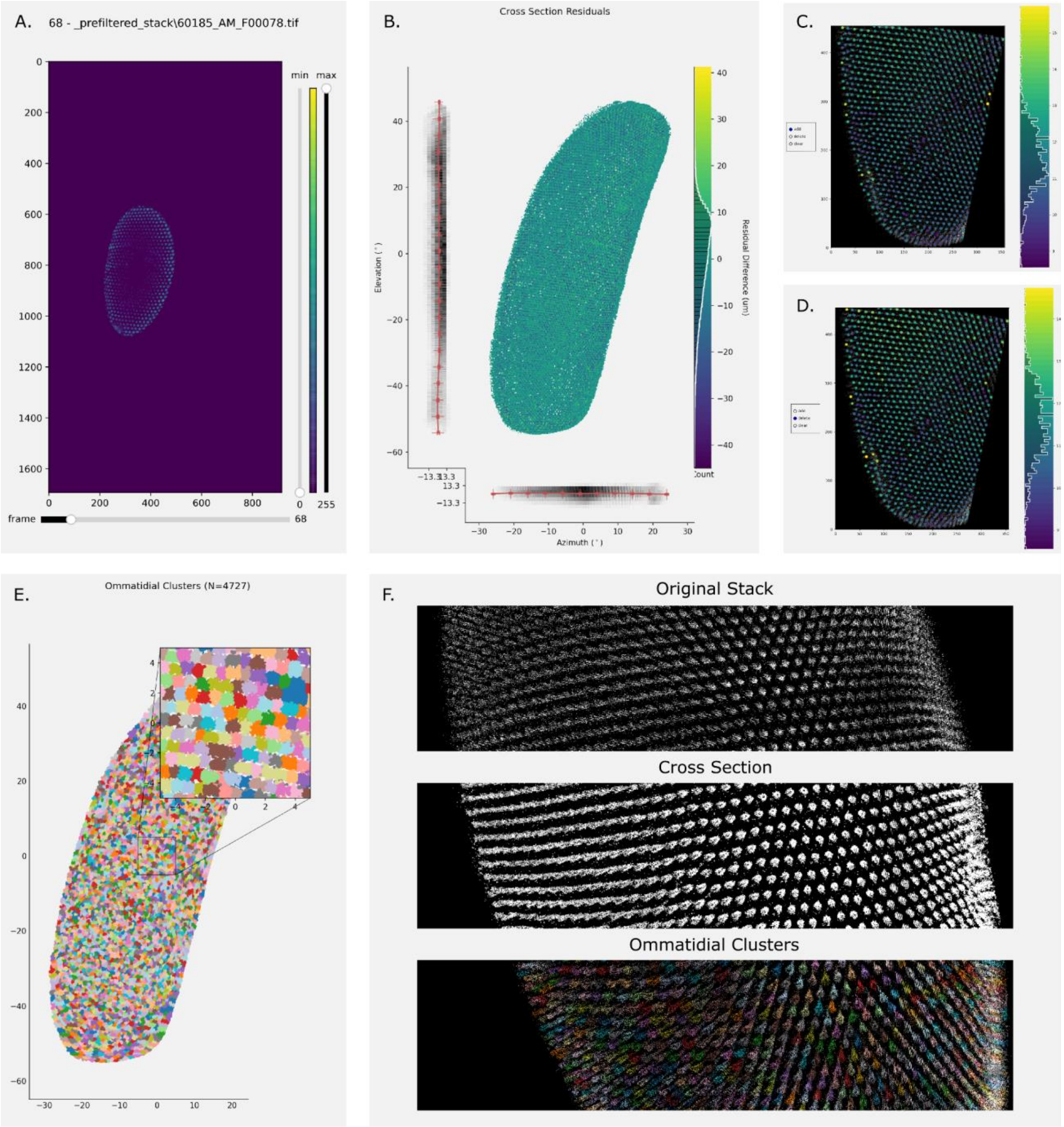

Supplementary Figure 1: **Graphical user interfaces for checking the progress of ODA-3D. a.** Layers of the stack can be displayed using the slider on the bottom and then prefiltered by selecting the lower and upper bounds of density measurements using the vertical sliders on the right. **b.** Once the thin surface is fit to the filtered voxels, the goodness of fit across the eye surface can be displayed. This allows the user to see if the surface was biased to any particular region. **c–d.** Once the ommatidial centers are approximated for a given segment of the eye, the user can add, delete, or clear all estimated centers. Each center point is colored based on its estimated diameter, so outliers are often highlighted by their extreme color. For instance, the user can identify and delete the two largest points (white arrows) in the top right of c. producing the frame in d. with an updated colormap. **e.** Then, the user can check the success of applying those centers to segment individual crystalline cones, each colored with a random color to assist in

comparisons. **f.** In addition, the user can view the outcome of each stage in an interactive 3D interface at the end of the procedure.
